# TRPV1 Potentiates TGFβ-Induction of Corneal Myofibroblast Development through an Oxidative Stress-Mediated p38-SMAD2 Signaling Loop

**DOI:** 10.1371/journal.pone.0077300

**Published:** 2013-10-02

**Authors:** Yuanquan Yang, Zheng Wang, Hua Yang, Lingyan Wang, Stephanie R. Gillespie, J. Mario Wolosin, Audrey M. Bernstein, Peter S. Reinach

**Affiliations:** 1 Department of Biological Sciences, State University of New York, State College of Optometry, New York, New York, United States of America; 2 Department of Ophthalmology, Icahn School of Medicine at Mount Sinai, New York, New York, United States of America; Wayne State University, United States of America

## Abstract

Injuring mouse corneas with alkali causes myofibroblast expression leading to tissue opacification. However, in transient receptor potential vanilloid 1 channel (TRPV1-/-) knockout mice healing results in transparency restoration. Since TGFβ is the primary inducer of the myofibroblast phenotype, we examined the mechanism by which TRPV1 affects TGFβ-induced myofibroblast development. Experiments were performed in pig corneas and human corneal fibroblasts (HCFs). Immunohistochemical staining of α-smooth muscle actin (α-SMA) stress fibers was used to visualize myofibroblasts. Protein and phosphoprotein were determined by Western blotting. siRNA transfection silenced TRPV1 gene expression. Flow cytometry with a reactive oxygen species (ROS) reporting dye analyzed intracellular ROS. [Ca2+]I was measured by loading HCF with fura2. In organ cultured corneas, the TRPV1 antagonist capsazepine drastically reduced by 75% wound-induced myofibroblast development. In HCF cell culture, TGF-β1 elicited rapid increases in Ca2+ influx, phosphorylation of SMAD2 and MAPKs (ERK1/2, JNK1/2 and p38), ROS generation and, after 72 hrs myofibroblast development. SMAD2 and p38 activation continued for more than 16 h, whereas p-ERK1/2 and p-JNK1/2 waned within 90 min. The long-lived SMAD2 activation was dependent on activated p38 and vice versa, and it was essential to generate a > 13-fold increase in α-SMA protein and a fully developed myofibroblast phenotype. These later changes were markedly reduced by inhibition of TRPV1 or reduction of the ROS generation rate. Taken together our results indicate that in corneal derived fibroblasts, TGFβ- induced myofibroblast development is highly dependent on a positive feedback loop where p-SMAD2-induced ROS activates TRPV1, TRPV1 causes activation of p38, the latter in turn further enhances the activation of SMAD2 to establish a recurrent loop that greatly extends the residency of the activated state of SMAD2 that drives myofibroblast development.

## Introduction

Upon corneal stromal wounding, TGF-β1 and interleukins are secreted by the epithelium into the exposed stroma to induce apoptosis of keratocytes at the wound margin [1]. Afterwards, the wound becomes repopulated by resident keratocyte-derived fibroblasts and by bone marrow derived fibrocytes [2,3]. Induced by epithelium-derived TGF-β1 and other factors, the wound-localized keratocytes and activated fibroblasts change into non-motile α-SMA fiber-rich myofibroblasts that are able to exert contractile forces on the surrounding matrix as well as increase extracellular matrix (ECM) elaboration. While these forces are important to ensure rapid closure of the wound, local persistence of myofibroblasts leads to excessive secretion of fibrotic matrix and excessive tissue contraction causing scarring and/or tissue opacification.

A recent report demonstrated that activation by injury of transient receptor vanilloid type 1 (TRPV1) nonselective ion channels also contributes to determining the wound-healing outcome. Its involvement is apparent since in a TRPV1^-/-^ mouse [4] the wound healing response to an alkali burn resulted in restoration of corneal transparency rather than opacification. Furthermore, the fact that myofibroblasts were not observed in the healed wound may suggest that TRPV1 inhibitors block TGF-β1-induced myofibroblast formation. We recently identified functional TRPV1 expression in human corneal fibroblasts (HCF) [5], but its role in mediating fibrogenic responses to TGF-β1 has not yet been established.

TGF-β1 plays an essential role in the wound healing associated fibroblast to myofibroblast transition in multiple tissues, including the cornea. In many instances these phenomena have been shown to involve SMAD2/3 and p38 MAPK pathways [6]. In addition, myofibroblast different has been shown to be dependent on reactive oxygen species (ROS) generated through NADPH oxidases (NOXs) [7]. Although functional expression of Nox1, 4, 5 has been recently reported in HCFs [8] their role in mediating TGF-β1 linked signaling events has not been determined.

We now show that a) TGF-β1-induced accumulation of α-SMA and development of a myofibroblast phenotype requires prolonged activation of p-SMAD2; b) these closely related phenomena are highly dependent on TRPV1 activity; c) stimulation by TGF-β1 of its cognate receptor, TGFβR, elicits TRPV1 activation through ROS formation; d) activated TRPV1 results in activation of p38 MAPK, which in turn sustains the initial SMAD2 activation. This results in a positive feedback loop that extends SMAD2 activation, augmenting the subsequent degree of α-SMA accumulation that characterizes the myofibroblast phenotype.

## Materials and Methods

### Cell culture and reagents

Human cadaver corneas from unidentifiable diseased subjects were obtained from The National Disease Research Interchange (http://ndriresource.org/). The Icahn School of Medicine Institutional Review Board has informed us in writing that, as described under section 45 CFR Part 46 of the U.S.A. Code of Federal Regulations unidentifiable cadaver tissue does not constitute research in Human subjects (see http://grants.nih.gov/grants/policy/hs/faqs_specimens.htm?Display=Graphics for further information). Hence, the experiments performed in this report do not require their approval or waiver. Stromal keratocytes were isolated as previously described [9]. Fresh keratocytes were cultured in Dulbecco’s Modified Eagle’s Medium/Ham’s Nutrient Mixture F-12 (DMEM/F12) plus 10% FBS (Atlas Biologicals, Fort Collins, CO) for up to eight passages [10]. In this media, keratocytes differentiate into human corneal fibroblasts (HCF). Culture medium and fetal bovine serum (FBS) were Gibco brand (Invitrogen, Grand Island, NY). For cell culture experiments with HCFs, cells were plated in DMEM/F12 plus 10% FBS. After 24 h medium was switched to supplemented serum-free medium (SSFM; DMEM/F12 plus 1X RPMI-1640 Vitamin Mix, 1X ITS Liquid medium supplement, 1 mg/ml glutathione, 2 mM L-glutamine, 1 mM sodium pyruvate, 0.1 mM MEM non-essential amino acids all purchased from Invitrogen, Grand Island, NY) with ABAM and gentamicin (Sigma, St. Louis, MO) for 24 h prior to experimentation. TGF-β1 (added from stock to a final 1 ng/ml) was from R&D Systems (Minneapolis, MN), the TRPV1 inhibitor capsazepine (CPZ), the TRPV1 agonist capsaicin (CAP), the TGFβRI-SMAD2/3 inhibitor SB431542, and the p-p38 inhibitor SB203580 were from Cayman Chemical Company (Ann Arbor, Michigan). The NOX inhibitor, diphenyleneiodonium (DPI) and the reactive oxygen species (ROS) inhibitor, N-acetylcysteine (NAC) were purchased from Sigma. Unless stated otherwise, these inhibitors were used at 10 µM.

### siRNA transfection

Small interfering RNA (siRNA) transfection was performed as described by the manufacturer (Santa Cruz Biotechnology, Santa Cruz, CA). Briefly, cells were grown in six-well plates to 40% confluence in antibiotic-free 10% FBS-containing medium. For each transfection, 6 µl TRPV1 siRNA (a pool of 3 different siRNA duplexes. sc-36826A: Sense: GAAGACCUGUCUGCUGAAAtt; Antisense: UUUCAGCAGACAGGUCUUCtt. sc-36826B: Sense: CGAGCAUGUACAAUGAGAUtt; Antisense: AUCUCAUUGUACAUGCUCGtt. sc-36826C: Sense: CGCAUCUUCUACUUCAACUtt; Antisense: AGUUGAAGUAGAAGAUGCGtt) was mixed with 6 µl of transfection reagent (sc-29528) in 200 µl transfection medium (sc-36868). Mixtures were incubated for 30 min at room temperature. Each well was washed once with 2 ml of transfection medium and then filled with one of the siRNA mixtures diluted in 8 ml transfection medium. After overnight incubation, an additional 1 ml of medium supplemented with 20% FBS was added to each well for another 24 h. This transfection mixture was removed and replaced with normal growth medium. All measurements were performed 72 h following transfection at which time TRPV1 levels decreased by 60-65% [5]. Scrambled siRNAs (sc-37007) were used as a control for monitoring non-sequence-specific effects.

### Corneal organ culture

Pig eyes were obtained from Pel Freeze (Rogers, AK). Ocular globes were dipped 3 times in 10% Povidone: 90% PBS and immediately washed in PBS. An epithelial-side 7 mm-wide, 0.03-0.04 mm-dip lamella was excised from the central cornea using a corneal trephine and a standard corneal surgery method. These wounded corneas or unwounded controls, including a 2-3 mm-wide scleral rim were resected from the eye globes, mounted on 1% agarose and 1 mg/ml bovine collagen (Purcol, Advanced Biomatrix, Poway, CA) and treated with CPZ or vehicle (DMSO) for the first 4 h post wounding. After washes, the corneas were maintained in organ culture medium (SSFM plus 1 mM Vitamin C) for up to 14 days with the central corneal zone maintained above the fluid level. The ocular surface was moistened daily and the culture medium was changed every other day.

### Immunohistochemistry and Immunofluorescence

Porcine corneas were fixed in formalin, embedded in paraffin embedding and sectioned at 7 µm thickness. Deparaffinized sections were microwaved for 5 min in citrate buffer (10 mM, pH 6.4) for antigen retrieval. Non-specific binding was blocked by 3% serum and α-SMA was immunodetected with a cy3-conjugated anti- α-SMA antibody (Sigma). HCFs were plated on coverslips in complete medium for 24 h, serum starved in SSFM for 24 h and then treated with 10 µM CPZ or vehicle (DMSO) for 1 h followed by addition of TGF-β1. After 72 h, cells were fixed in 3% paraformaldehyde for 15 min, permeabilized with 0.1% Triton X-100 and, after blocking with 3% normal mouse serum, were incubated with the anti-α-SMA-cy3 antibody. Sections were viewed with a Zeiss Axioskop microscope and images were captured at identical exposure times with a SPOT-2 CCD camera (Diagnostic Instruments, Sterling Heights, MI). Using ImageJ, a threshold pixel intensity was set and α-SMA positive cells were counted.

### Western Blot

Cells were gently washed twice in cold phosphate-buffered saline (PBS) and harvested in cell lysis buffer (20 mM Tris, 150 mM NaCl, 1 mM EDTA, 1 mM EGTA, 1% Triton X-100, 2.5 mM sodium pyrophosphate, 1 mM β-glycerol phosphate, and 1 mM Na _3_VO_4_, pH 7.5, 1 mM PMSF, 1 mM benzamidine, 10 µg/ml leupeptin, and 10 µg/ml aprotinin) and fully disrupted by sonication. After centrifugation at 13,000 rpm for 15 min, supernatant protein concentration was determined by the bicinchoninic acid assay (micro BCA protein assay kit; Pierce Biotechnology, Rockford, IL). Protein was denatured by boiling for 1 min and 10-50 µg protein was electrophoresed in 10% polyacrylamide sodium dodecylsulphate mini-gels and electroblotted onto PVDF membranes (Bio-Rad, Hercules, CA). Membranes were blocked with 5% fat-free milk in 1x PBS buffer-0.1% Tween-20 for 1 h incubated overnight at 4°C with primary antibodies and for 1 h at room temperature with an 1:2000 dilution of an indicated HRP-conjugated secondary antibody. Bound HRP activity was visualized using ECL Plus (GE Healthcare, Piscataway, NJ). Chemiluminescence was captured with X-ray film and quantized by densitometry using SigmaScan Pro (Sigma). All experiments were repeated at least three times unless otherwise indicated.

### ROS measurements

Cells were grown in six-well plates. After a 24 h incubation in serum-free medium, they were incubated for 15 min with 2 µM CM-H2DCFDA (Molecular Probes, Eugene, OR); a cell entrapable dye that develops fluorescence after oxidation prepared in Dulbecco’s modified PBS (DPBS) and subjected to various treatments as detailed in Results. After trypsinization, centrifugation and resuspension in DPBS buffer complemented with 1 µg/ml propidium iodide (MP Biomedical) to stain dead cells, the extent of fluorescent generated live cells was determined by flow cytometry in an Accuri 6 instrument (Accuri, Ann Arbor, MI) using a 488/531 nm excitation/emission combination. For the measurement of TGF-β1-induced responses at 16 h, dye loading was performed after rather than prior, to the pharmacological exposures.

### Cell fluorescence imaging for [Ca^2+^] determination

Cells grown on 22-mm diameter coverslips (Fisher Scientific, Pittsburgh, PA) were loaded with 2 µM fura-2AM (Molecular Probes, Eugene, OR) at 37°C for 30 min and then washed with NaCl Ringer’s solution containing (in mM): NaCl (141), KCl (4.2), CaCl_2_ (0.8), KH_2_PO_4_ (2), MgCl_2_ (1), glucose (5.5), and HEPES (10) with osmolarity 300 mOsm and pH 7.4. Following dye loading, single-cell fluorescence imaging was performed on the stage of an inverted microscope (Nikon Diaphot 200) using a chamber with a coverslip formed base. For experiments conducted under calcium-free conditions, cells were first pre-incubated for 10 min with a calcium-free counterpart supplemented with EGTA (2 mM). Cells were then alternately illuminated at 340 and 380 nm, and emission was monitored every 3 sec at 510 nm using a Roper Scientific CCD camera. Each field of interest contained 15~20 cells. Changes in [Ca^2+^]_i_, were analyzed using Ratio Tool software (Isee Imaging, Durham, NC).

### Data analysis

All results are reported as means ± SEM. Statistical significance was assessed using paired or non-paired Students t-test. The different levels of significance are designated as follows: *p value<0.05, **p value<0.01, ***p value<0.001.

## Results

### Inhibition of TRPV1 abolishes myofibroblast accumulation in a pig organ culture wound healing model

**Figure 1:**
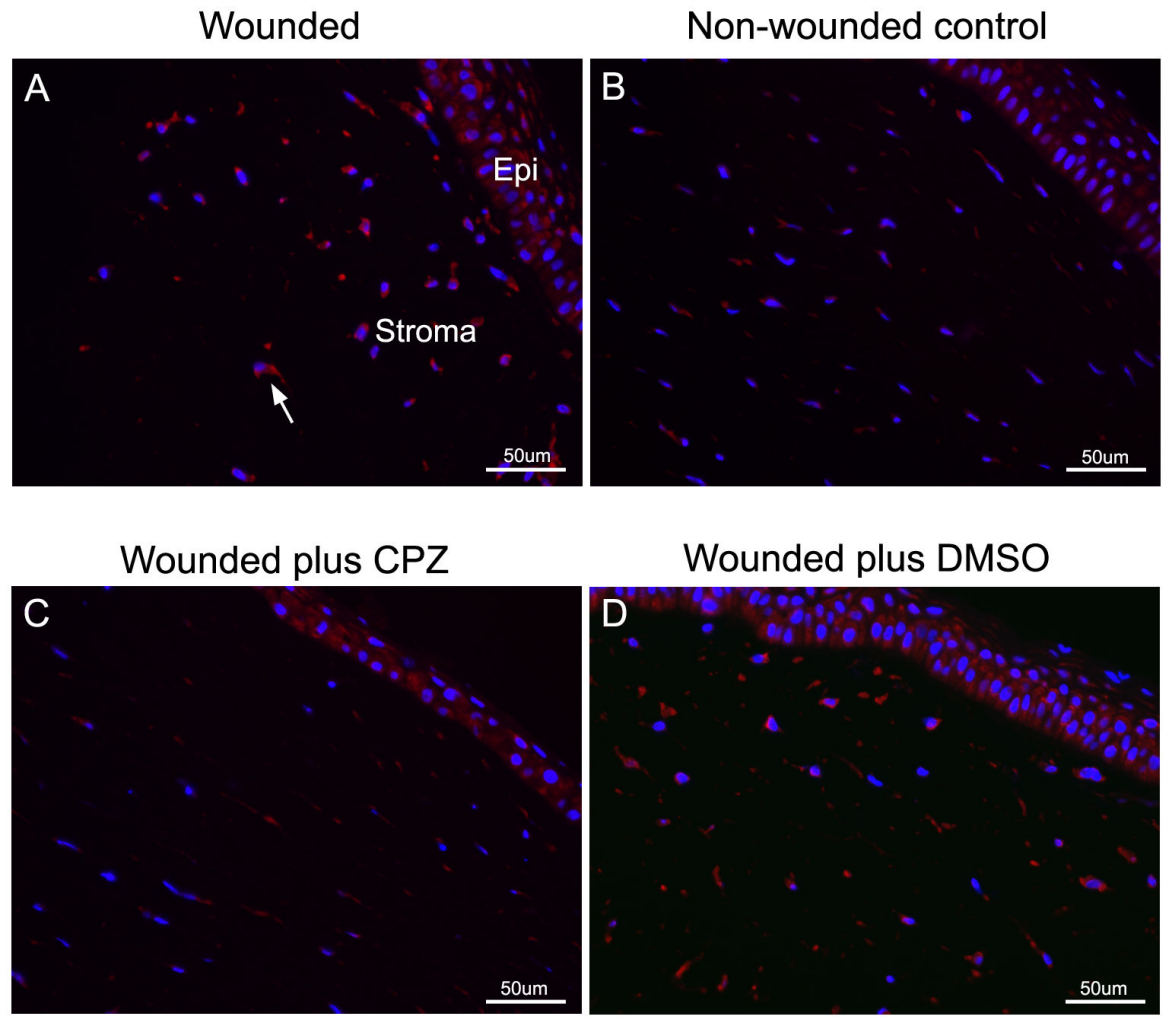
Effect of wounding and TRPV1 inhibition on myofibroblast presence in pig anterior corneal stroma after corneal surface healing. **A**. Wounded cornea. **B**. Non-wounded (control). **C** and **D**. Wounded corneas treated, respectively, with 10 µM CPZ in DMSO or DMSO alone (vehicle). Histological slides were generated and immunostained with antibody to α-SMA as described in Methods. Bar = 50µm. Representative experiment of three independent replicates completed.

To probe for the role of TRPV1 function in wound healing, we established an *ex-vivo* porcine organ culture model in which myofibroblasts are generated following an anterior keratectomy; in this wound the epithelium, the basement membrane, and approximately the top third of the corneal stroma is removed. During the subsequent organ culture healing period the epithelium, resurfaces the underlying wounded stroma and undergoes de novo stratification. The healing epithelium actively secretes TGF-β1and IL-1 into the underlying stroma. In several *in vivo* animal models, it has been shown that these secretions induce corneal fibroblast (keratocyte) apoptosis in the wound margin. This is followed by repopulation of the wound margin with keratocytes/fibroblasts derived from the resident stroma and from bone marrow-derived fibrocytes [3]. Finally, these cells develop into corneal myofibroblasts with profuse accumulation of α-SMA fibers [2]. Using the described keratectomy-organ culture approach, this procedure generated a 7.9 ± 2.7 fold (p<0.05) increase in myofibroblast expression (Figure 1A) compared to non-wounded tissue (Figure 1B). Incubation of the wounded surface with CPZ for the first 4 h post-wounding dramatically reduced by 75 ±11% (p<0.05). the development of myofibroblasts (Figure 1C) when compared with only the incubation inhibitor vehicle: (DMSO) (Figure 1D).

### Inhibition of TRPV1 blunts TGFβ1-induced myofibroblast development in corneal fibroblasts

**Figure 2:**
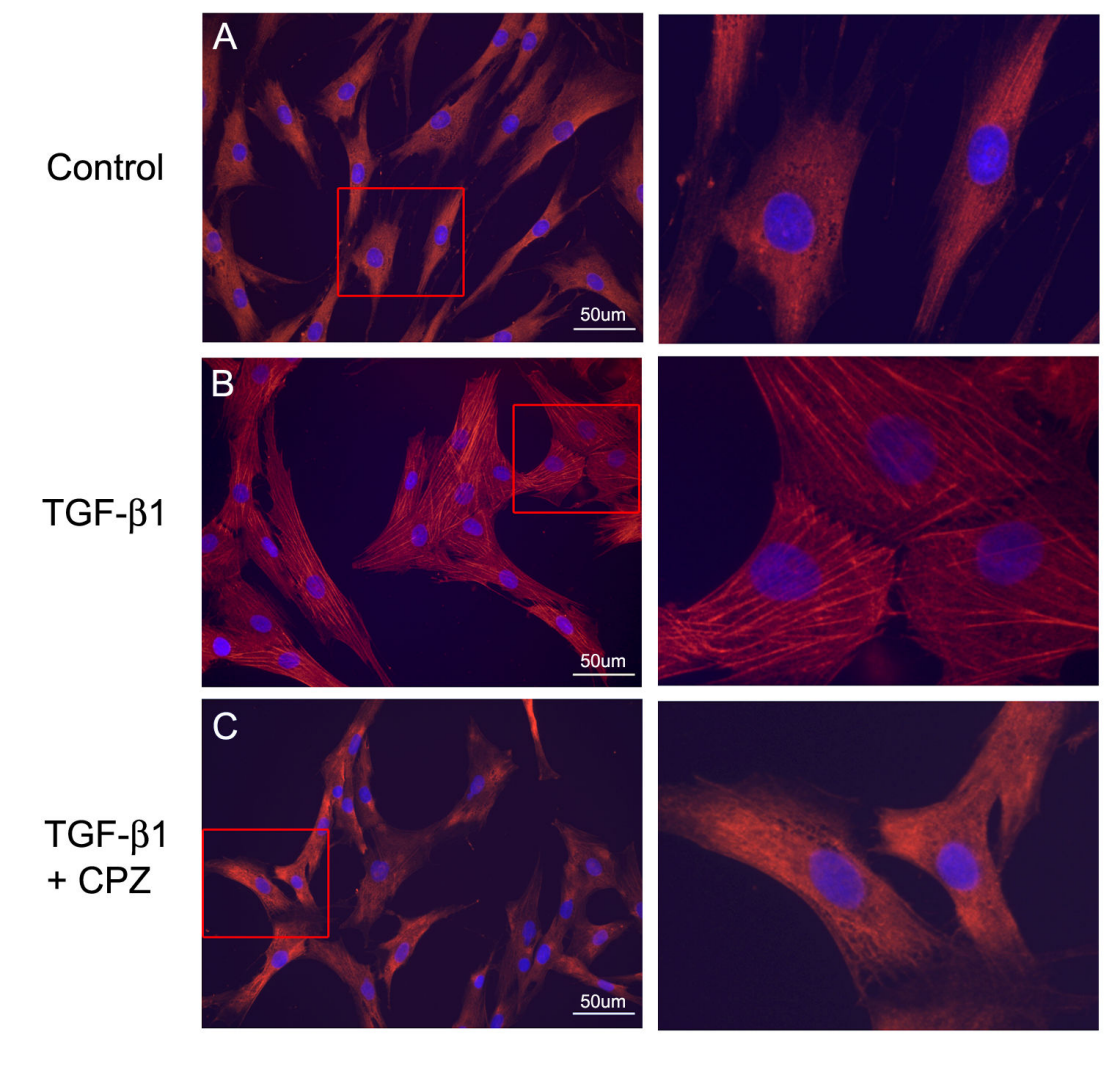
Effect of TRPV1 inhibition on myofibroblast development in culture. Seventy two hrs post-treatment images. **A**. Control. Note absence of stress fibers. **B**. TGF-β1. Prominent α-SMA fibers and cross sectional cell area increases are patent. **C**. Cells preincubated with CPZ prior to exposure to TGF-β1. Right panels are magnified view of framed areas in the left panels. Note the decrease in cross sectional cell area back towards the control and the drastic reduction in α-SMA fibers. Bar = 50µm.

To examine in detail the involvement of TRPV1 activity in the generation of myofibroblasts in the wounded human cornea, we utilized primary cultures of fibroblasts derived from corneal keratocytes, a system that is both more amenable to experimentation than the whole cornea and can be implemented using cells from a human source [10,11,12,13]. As previously described [10,14], a 72 h incubation of these cells in TGF-β1-complemented culture medium resulted in patent development of α-SMA-containing stress fibers (Figure 2A and B) and consistent with, the results of Okada et al [4] in mice and the results above in pig, the presence of the TRPV1 inhibitor CPZ led to a major reduction of the extent of this development as evidenced by the inhibition of α-SMA containing stress fibers (Figure 2C).

### TRPV1 silencing reduces SMAD2 activation and α-SMA accumulation

**Figure 3:**
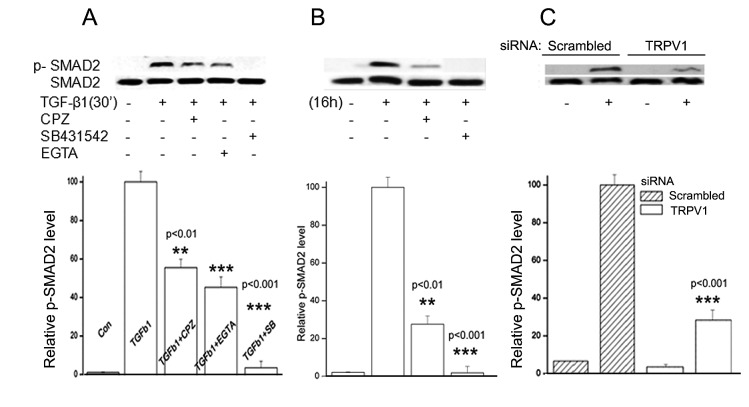
Dependence of TGF-β1 induced Smad2 phosphorylation on TRPV1 activation. Cells were treated with, DMSO, CPZ or SB431542 for 60 min or 2 mM EGTA for 15 min prior to exposure to TGF-β1. SMAD2 and p-SMAD2 were detected in Western blots. **A**. Cells were lysed after 30 min. **B**. Cells were lysed after 16 h. **C**. HCFs were transfected with either scrambled siRNA or TRPV1 siRNA and after 24 h were treated with TGF-β1 for 30 min. Graphs represent the average and standard error ratios p-SMAD2/SMAD2 ratios of three independent experiments, relative to the ratios obtained in the TGF-β1-only experiments, which have been arbitrarily set at 100%.

Upon TGF-β1 binding to its cognate receptor (TGF-βR), receptor-bound transcription factors SMAD 2/3 are phosphorylated by intrinsic receptor kinase activities [15]. Activated SMAD2/3 translocate to the nuclei leading to modified gene expression. In the HCFs, TGF-β1 induced an early response increase in p-SMAD2 over baseline value within the first 30 min (Figure 3A). Pre-treatment with CPZ (Figure 3A) or TRPV1 gene silencing (Figure 3C) [5] resulted in a drastic reduction in the degree of SMAD2 phosphorylation. Bathing the cells in Ca^2+-^-sequestering medium, caused a larger reduction of the response to TGF-β1 than that observed with CPZ, consistent with the notion that TRPV1 effects are fully dependent on activation of a cell membrane calcium channel [16]. As should be expected, pre-incubation with a TGF-βR-SMAD2/3 inhibitor (SB431542), completely blocked the response to TGF-β1 (Figure 3A).

A second set of experiments where TGF-β1 application was extended to 16 h was also completed (Figure 3B). The continuous presence of TGF-β1 resulted in a continuously increasing p-SMAD2 level, and, similar to the early response phase, CPZ treatment resulted in marked reduction of SMAD2 (Figure 3B). Overall, the results depicted in these panels show that, preventing the TRPV1 calcium channel function leads to a marked reduction in the extent of SMAD2 phosphorylation.

**Figure 4:**
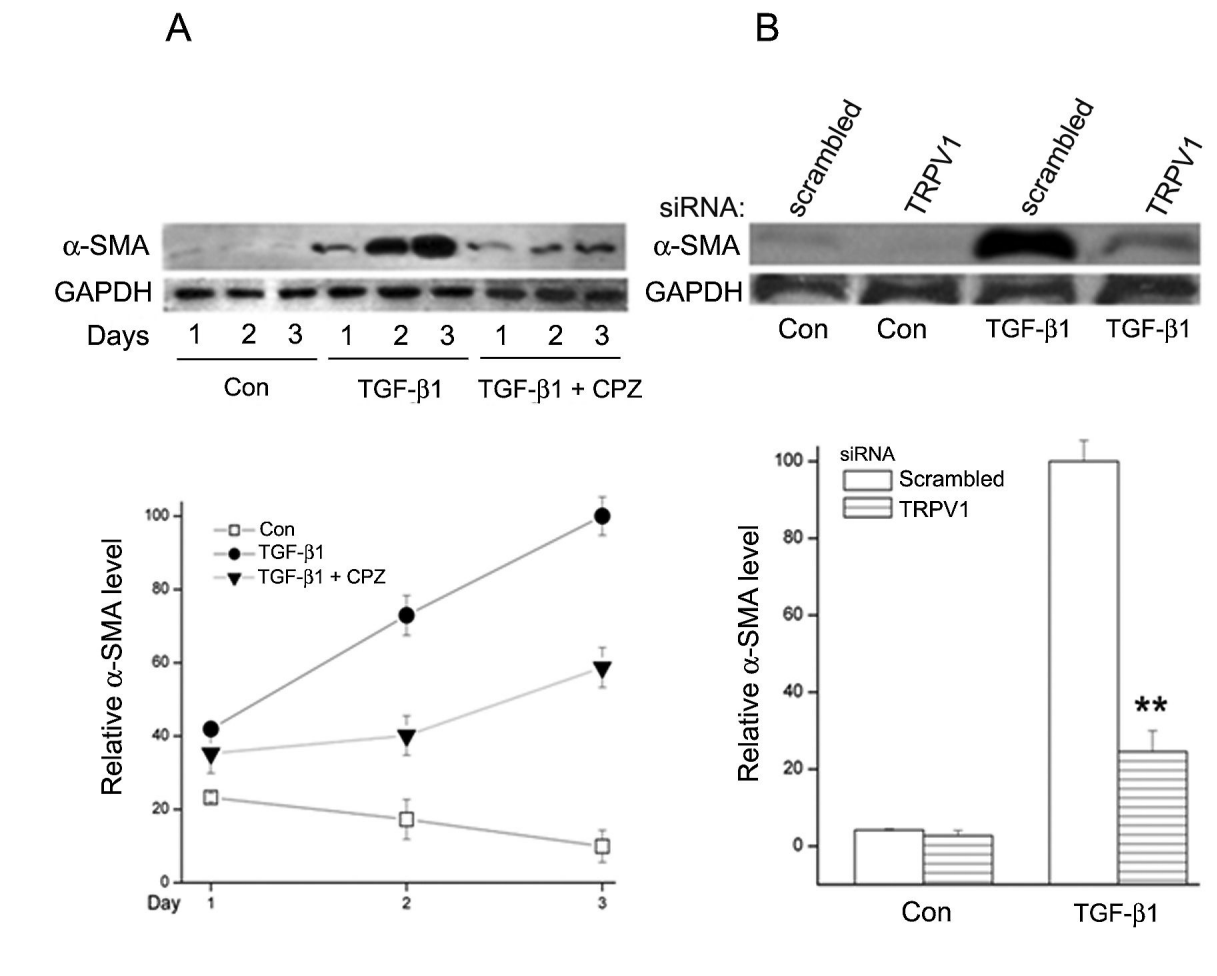
TGF-β1 effect on α-SMA protein level and effect of TRPV1 inhibition as a function of HFC culture time at 72h. A. TGF-β1 causes a large increase in α-SMA protein which is markedly reduced by the presence of CPZ in the culture medium. **B**. Effect of TRPV1 siRNA gene silencing; the reduction of α-SMA accumulation is similar to that achieved with CPZ. Averages and standard errors for three independent experiments are shown.

Concomitant with the activation of SMAD2, TGF-β1 caused a large increase in α-SMA protein. This rise was a slow process that extended well beyond the typical time course of the signal transduction events that follow TGF-β1 addition (Figure 4A). Under continuous exposure to TGF-β1, the α-SMA level increased about 4 times after two days and 18 times by day three. Co-incubation of TGF-β1with CPZ limited the α-SMA rise on day 3 to less than 30% of the increase caused by TGF-β1 alone. Gene silencing by the TRPV1 siRNA had an inhibitory effect comparable to that caused by CPZ (Figure 4B).

### TGF-β1-induces CPZ sensitive calcium influx

**Figure 5:**
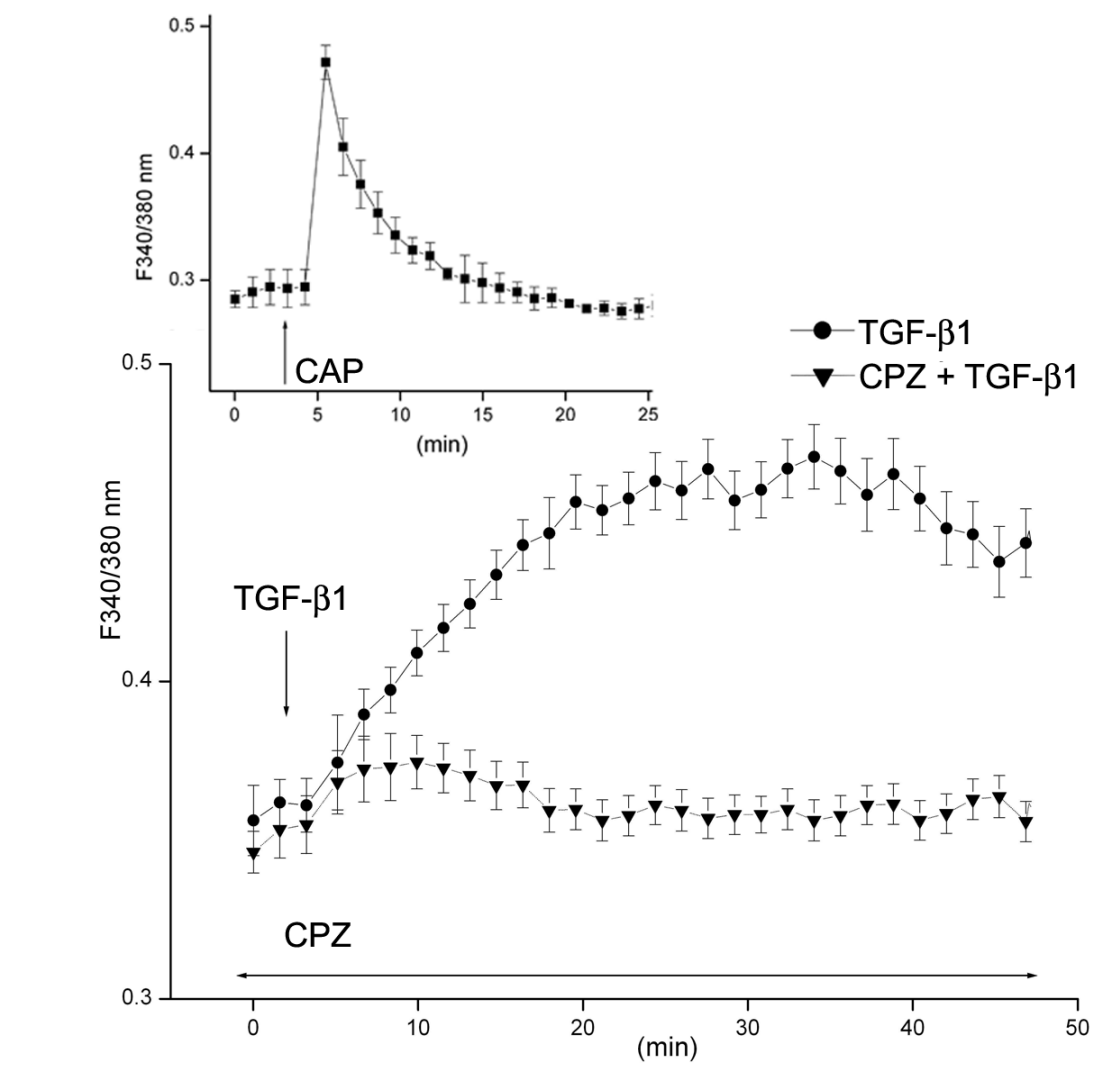
TGF-β1 induces Ca^2+^ influx through TRPV1 activation. Fura-2AM-loaded HCF were monitored for changes in [Ca^2+^]_i_ using single cell imaging. HCFs were exposed to either CAP or TGF-β1 with or without the presence of CPZ, as indicated. Averages and standard errors of three independent experiments are shown.

TRPV1 activity is intimately associated with cytosolic calcium influx [5,17]. In HCFs the agonist CAP causes a very rapid rise in [Ca^2+^]_i_ that can be completely cancelled by preincubation with CPZ or removal of extracellular calcium (Figure 5) Since CPZ suppressed TGFβR-linked signaling responses (Figures 1-4), we examined whether a rise in intracellular Ca^2+^ was also a component of TGF-β1 signaling. Figure 5 demonstrates that both TGF-β1and CAP induced CPZ-sensitive intracellular Ca^2+^ rises. These two calcium transients, though, differed markedly in their time courses; a peak [Ca^2+^]_i_ increase with the TRPV1 agonist occurred within one min; the maximal level attained with TGF-β1 occurred at about 25 min and displayed a very slow post peak decrease.

### TGFβ1 selectively induces prolonged p38 activation in a TRPV1 dependent manner

**Figure 6:**
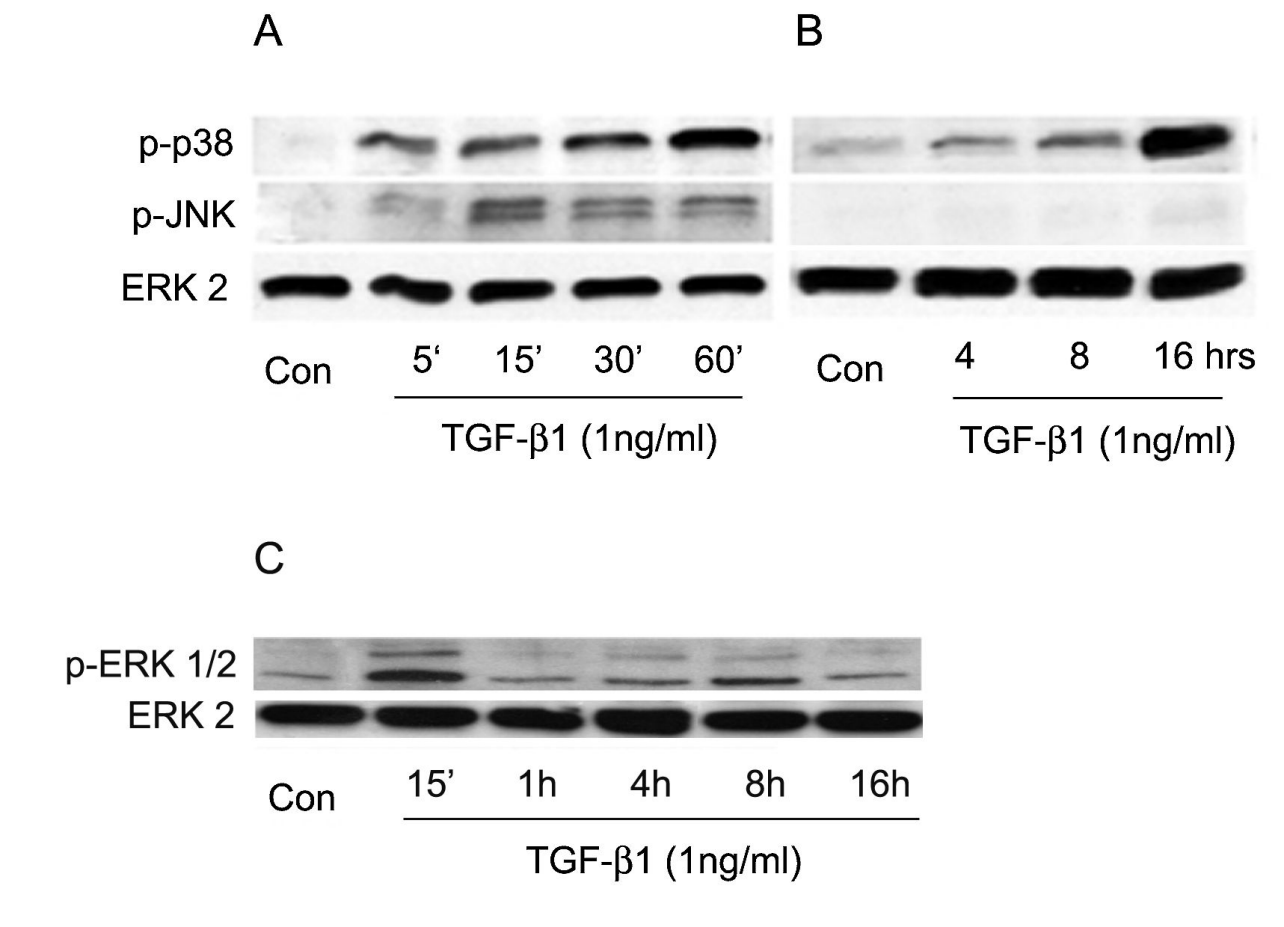
Effect of TGF-β1 on phosphorylation of MAPKs. **A**. Phosphorylation of p38 and JNK over the first 60 min following addition of TGF-β1. **B**. Phosphorylation time course over 16 h. C.A continuous time course was performed for p-ERK. Similar to JNK, ERK activation waned after early activation. Of all three activated MAPKs, activation persists only for p38. Total ERK2 was used as loading control. Results shown are representative of three independent experiments performed.

In addition to its effect on SMAD2/3, TGF-β1 also activates the MAPK pathway [15,18]. Accordingly we also examined how TGFβ1 exposure affects the phosphorylation status of the three final kinases of the MAPK cascades, p38, JNK1/2, and ERK1/2, over early to intermediate (0-60 min) and late (4-16 h) response phases. All three kinases exhibit enhanced phosphorylation by 5-15 min of TGF-β1 addition (Figure 6A-C). Over the following 60 min, the activation of JNK1/2 and ERK1/2 gradually decreased towards their pre-exposure values (Figure 6A and C). p38 activation, in contrast, was maintained and, in fact, the p- p38 level increased gradually over the subsequent 16 hrs (Figure 6B).

### Inhibition of p-p38 kinase reduces TGF-β1 -induced early and late SMAD2 phosphorylation and α-SMA accumulation

**Figure 7:**
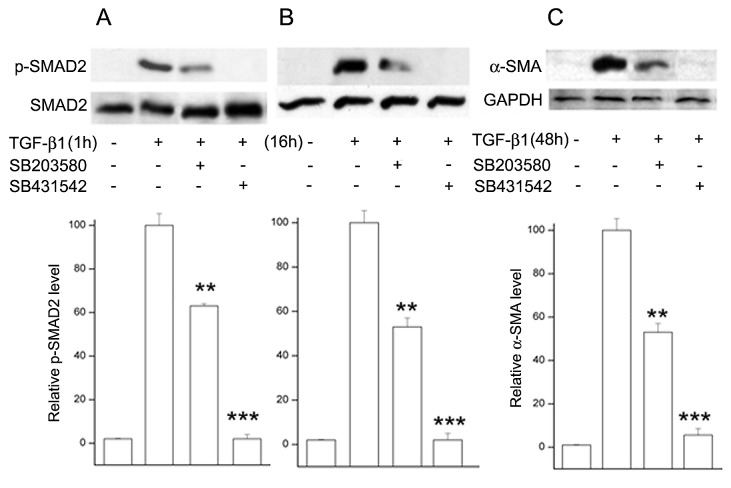
Effect of activated p38 on SMAD2 phosphorylation and α-SMA accumulation in HCFs incubated with TGF-β1 for various lengths of time. Where indicated, the p-p38 inhibitor SB203580 or the TGFβR-SMAD2/3 inhibitor SB431542 was added in the medium 1 h prior to introduction of TGF-β1. Cells were lysed after 1 h (**A**), 16 h (**B**) or 48 h (**C**). Lysates were subjected to Western blot analysis for p-SMAD2 or α-SMA. In the case of SMAD2 the membranes were stripped and re-probed for total-SMAD2. For α-SMA the loading control was GAPDH. Graphs represent the average and standard error ratios p-SMAD2/SMAD2 and α-SMA/GAPDH ratios of three independent experiments. Samples with TGFβ and without inhibitors were arbitrarily set at 100%.

The results above indicate that the persistent TGFβ-induced increases in p38 and SMAD2 phosphorylation are not shared by the other two final MAPKs, accordingly we addressed next the possibility that the activation of p38 and SMAD2 maybe linked or related. We found that HCF preincubation with the selective p-p38 inhibitor, SB203580 [19] caused large decreases in the TGFβ-induced p-SMAD2 levels at both 1 and 16 h (37% and 47%, respectively; Figure 7A and B). Blocking p-p38 activity also reduced the degree of the 3-day α-SMA accumulation by 48% (Figure 7C). As expected pre-treatment with the selective TGFβR-SMAD2/3 pathway inhibitor [20] also prevented SMAD2 phosphorylation and α-SMA accumulation***.***

### TGFβ1-induces ROS formation in HCF with the involvement of TRPV1

**Figure 8:**
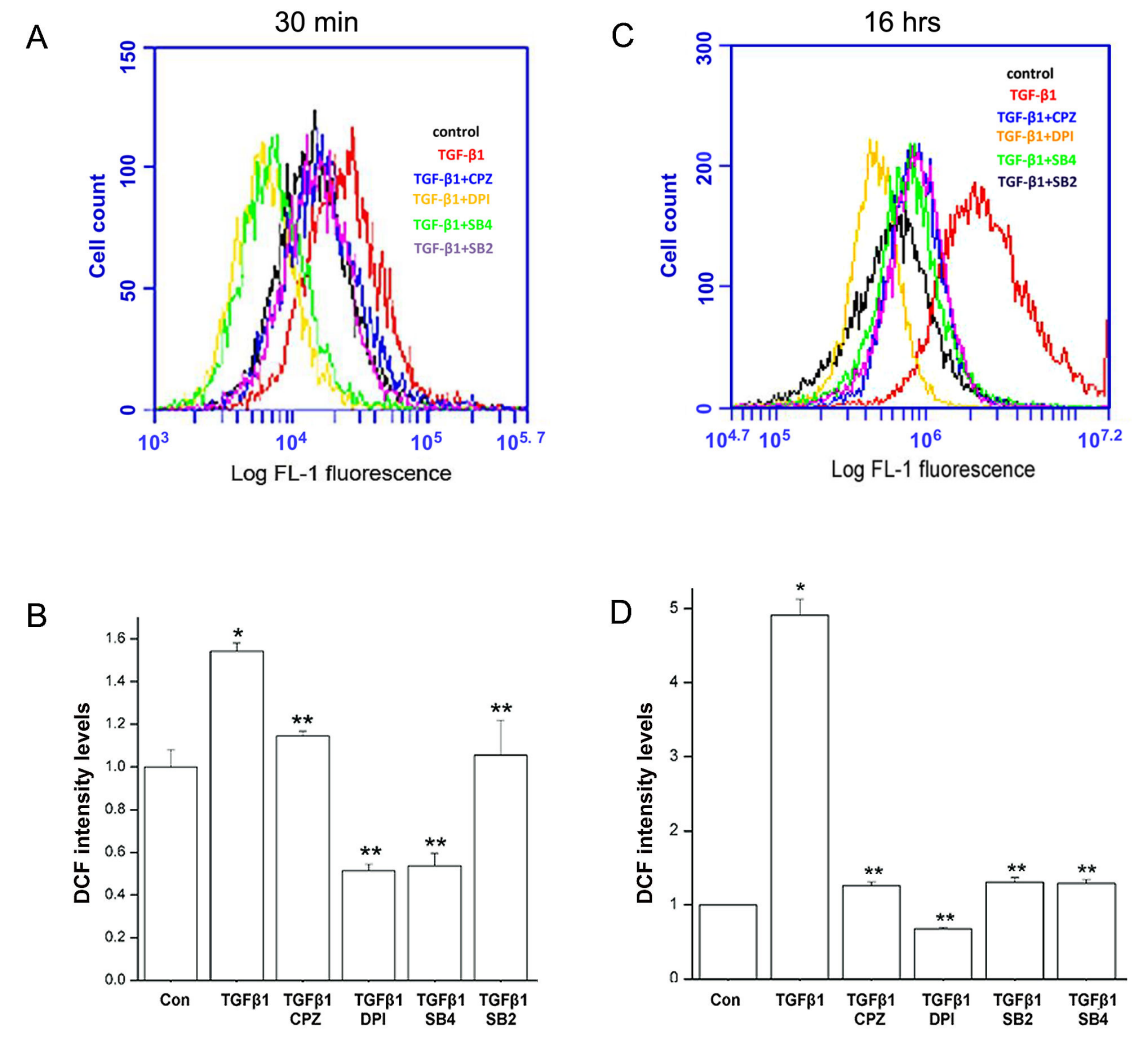
Effect of TGF-β1 on ROS activity in HCFs. **A**. Effects after 30 min incubation with the growth factor in control condition or with pretreatments with CPZ, SB431542, or SB203580, all for 1 h or DPI for 2 h. **B**. Equivalent results when the incubation was continued for 16 h after addition of TGF-β. Summary histograms of DCF fluorescein intensity depicting averages and standard error fold changes from the control values of three independent experiments are also shown.

Emerging evidence suggests that ROS controls a plethora of pathophysiological responses to TGF-β1, including the fibroblast to myofibroblast transition [21,22]. Increases in ROS level can be detected soon after TGFβ introduction and for up to 24 hrs. Here, we used the oxidation sensitive fluorescent dye CM-H2DCFDA to obtain relative measurements of ROS levels in the HCFs. Measurements were performed at either 30 min or 16 hrs after addition of TGF-β1. An early response ROS generation was reached within 30 min of the addition of TGF-β1 (Figure 8A). At 30 min, dye oxidation rate in treated cells was 55% higher than in untreated controls whereas after 16 hrs this rate was 315% larger (Figure 8A and B), suggesting an increment of activity similar in its time course to that observed for the formation of p-SMAD2 (Figure 7). The ROS increases were nearly completely suppressed by pretreatment of the cells with the TGFβR-SMAD2 inhibitor, SB431542 and the NADPH oxidase (NOX) inhibitor, diphenyleneiodonium chloride (DPI) as would be expected [23], but they were also substantially inhibited by SB203580, the p-p38 inhibitor and by CPZ.

### TGF-β1 activation of TRPV1 calcium channel function is mediated by ROS

**Figure 9:**
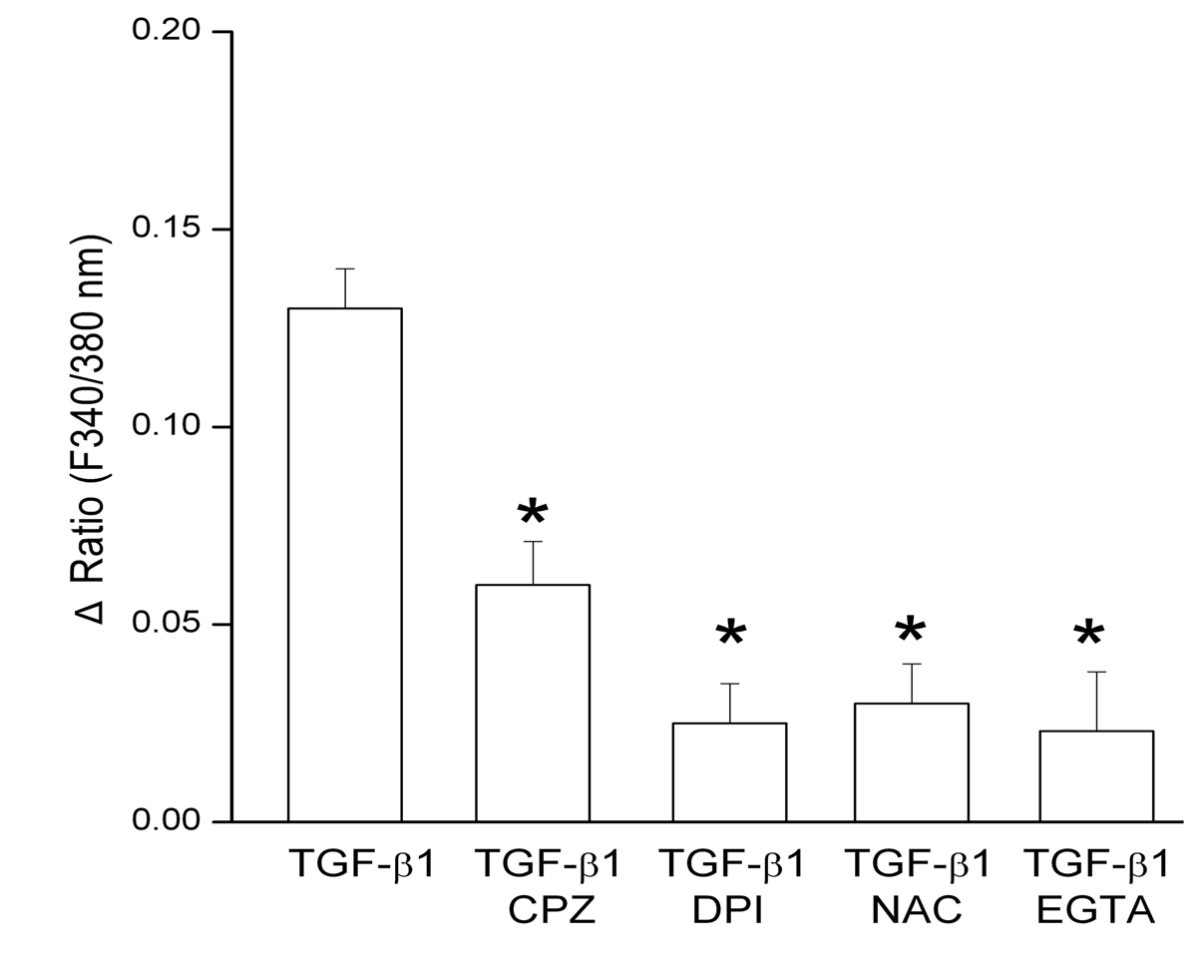
Effect of inhibitors of ROS generation on the TGF-β1-induced intracellular calcium rise. HCFs were exposed to TGF-β1 and relative changes in intracellular calcium were evaluated based on fura 2 image analysis as described in Methods. Summary histograms from three independent experiments are shown. *p<0.05 for inhibitor treated vs. control (TGF-β1 alone).

First, to expand our understanding of the signaling events involved in the dependence of the TGF-β1-induction of myofibroblast phenotype on TRPV1 function we tested if ROS activates TRPV1 thereby establishing a potentially self- sustaining activation loop. Such loops were described in multiple biological systems including induction of myofibroblasts by TGFβ [24,25,26,27,28]. We tested if inhibiting TGFβ1-induced ROS generation would prevent calcium influx. The results showed that indeed, ROS generation inhibition by either DPI or NAC suppressed TGF-β1 induced Ca^2+^ influx by 84% (Figure 9). Furthermore the [Ca^2+^]_i_ increase was also inhibited by extracellular calcium sequestration with EGTA demonstrating that it was due to calcium influx down its extracellular to cytosol gradient. CPZ also inhibited calcium influx validating that it was primarily mediated by TRPV1, though the remaining influx in this case leaves open the possibility of a minor involvement by other cell membrane or intracellular organelle calcium channels.

### TGF β-induced accumulation of α-SMA and phosphorylation of p38 and SMAD2 are dependent on ROS

**Figure 10:**
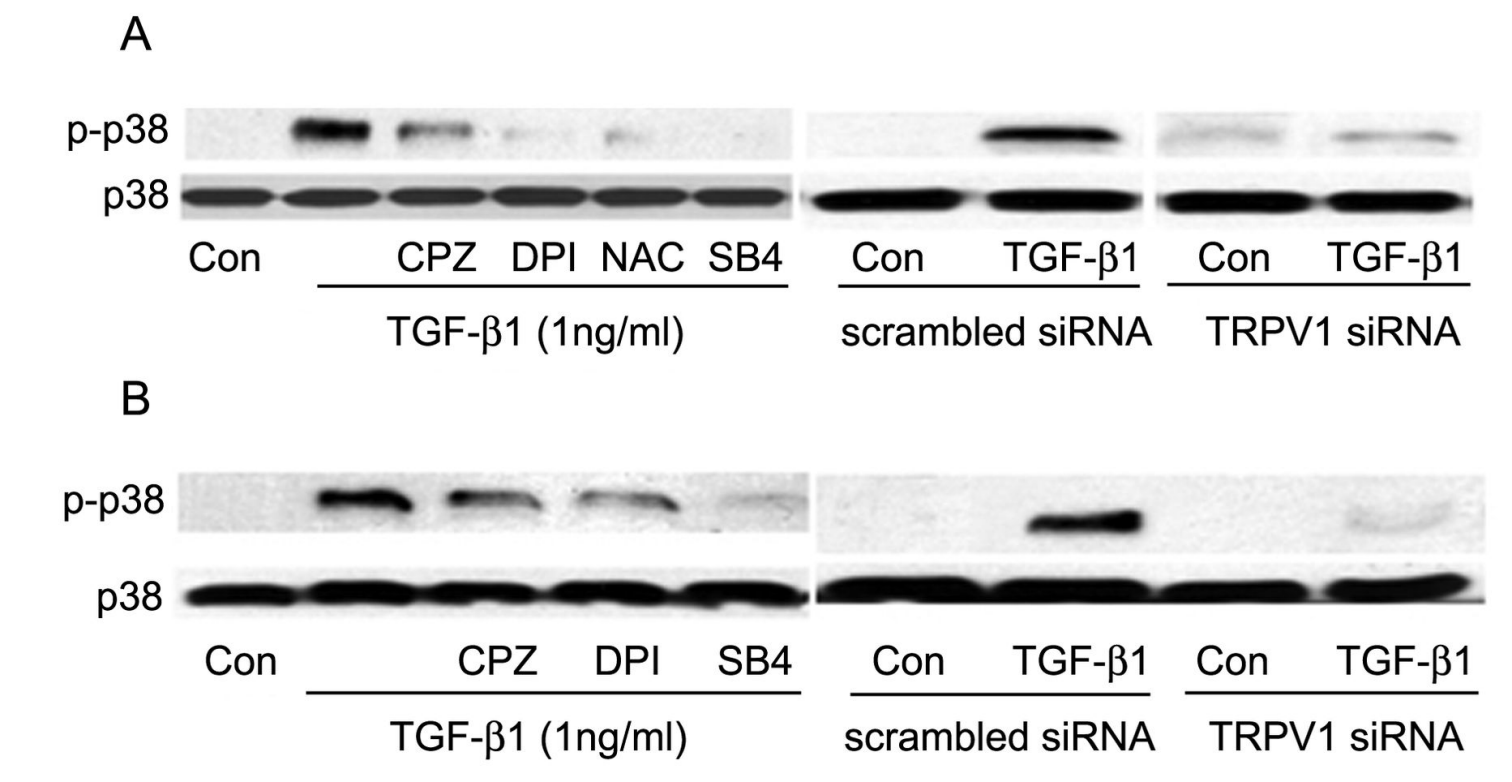
Effect of inhibition of TRPV1 activity and ROS generation on the early and late p38 phosphorylation response to TGF-β1. **A**. **Left**: Cells were pre-incubated for 15 min with 10µM CPZ, NAC, SB431542 (SB4) or for 2 h with DPI prior to exposure to TGF-β1 for 1 h with or without inhibitors. Cell lysates were Western blotted for p-p38 and p38. All inhibitors reduced p38 activation. **Right**: equivalent results in cells transfected with TRPV1 siRNA. **B**. **Left and Right**: Similar to A, but TGF-β1 treatment was extended to 16 h. Results shown are representative of three independent experiments performed.

Activation of TRPV1 by CAP resulted in phosphorylation of p38 and, as indicated by the calcium influx measurements, ROS accumulation mediated TGF-β1 induction of TRPV1 activity. Accordingly, we next examined whether the effect of ROS on the TGF-β1 -induced TRPV1 function extended to p-p38 accumulation. As described in Figure 6, the p-p38 levels, which were nearly null for all practical purposes prior to introduction of TGF-β1, were easily detectable at both 1 and 16 h post-TGF-β1 addition. This p-p38 accumulation was drastically reduced by the ROS inhibitors, by CPZ or pretreatment with the siRNA for suppression TRPV1 gene expression (Figure 10). By themselves, CPZ, DPI, NAC or SB431542 did not elicit changes in the very low baseline p-p38 level (data not shown).

**Figure 11:**
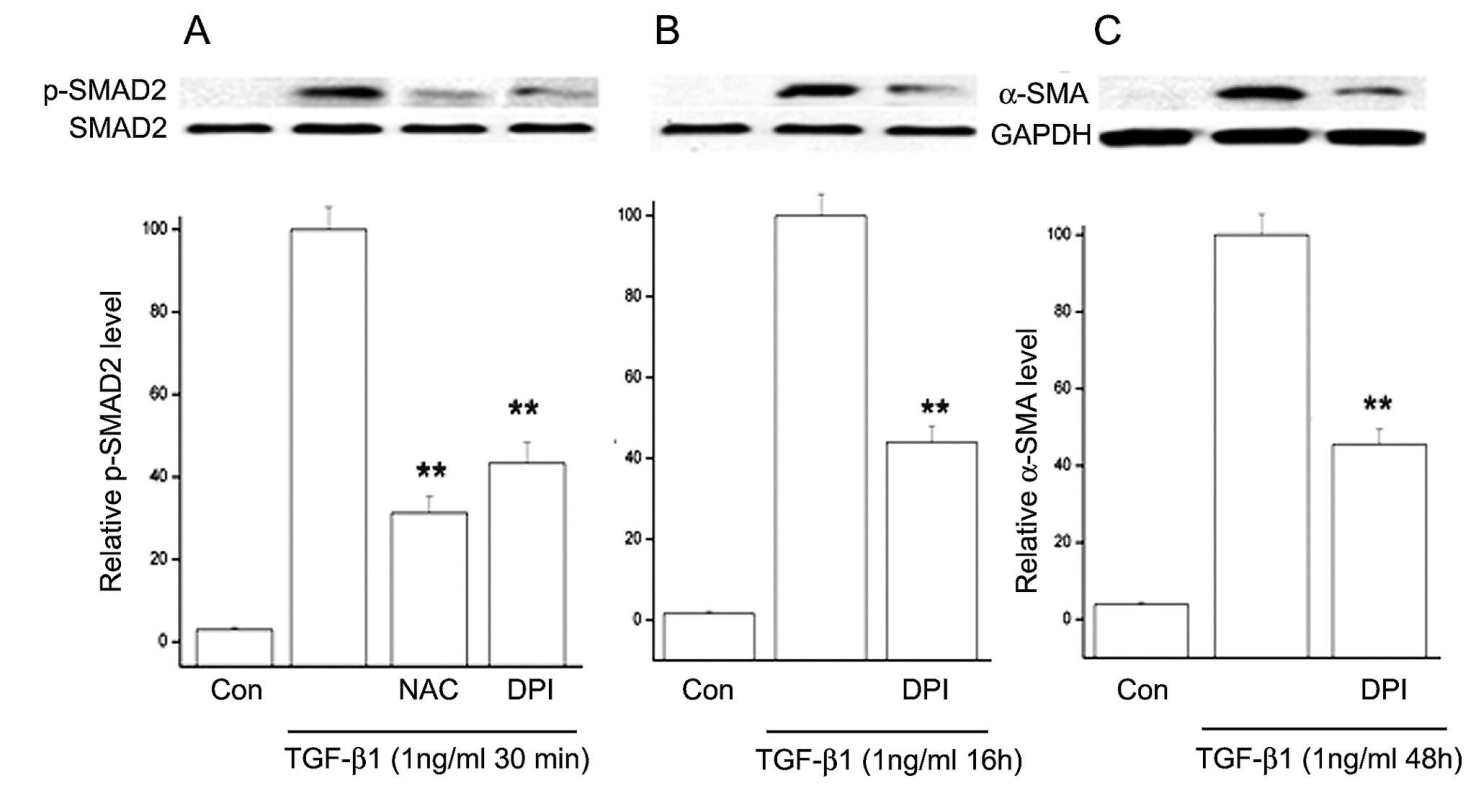
Effect of inhibitors of ROS generation on TGF-β1-induced SMAD2 phosphorylation and α-SMA expression. **A**. p-SMAD2 levels 30 min after introduction of TGF-β1 with or without prior addition of NAC or DPI. **B**. Equivalent results when the incubation with TGF-β1 was extended to 16 h. **C**. α-SMA accumulation after 48 h incubation with TGF-β1 witouth or without DPI. Averages and standard errors of three independent experiments are shown Values for the cells treated only with TGF-β1 have been set at 100%.

Finally, we reasoned that, since the results presented above demonstrated that a long-lived high p-SMAD2 level and the continuous accumulation of α-SMA over a three day period depend on a continuously active p38, inhibition of ROS should also affect these activities. Validating the stated relationships, DPI or NAC reduced TGF-β1-induced SMAD2 phosphorylation and α-SMA accumulation by 50-70% (Figure 11). Although this inhibition is significant, it was smaller than the near complete inhibition of the activities or parameters directly associated with TRPV1 function, suggesting that direct inhibition of TRPV1 is a more effective approach to quenching the above described pathways.

## Discussion

In cultured fibroblasts from various sources, TGF-β induces transition to the myofibroblast phenotype [10,11,12,13,14,29,30,31]. Understanding this conversion is key to controlling fibrosis since it is the persistence of myofibroblasts in healing tissues that leads to excessive matrix accumulation, over-contracture and fibrotic scarring. Our current study shows that in the wounded cornea and in the fibroblasts derived from this tissue, the magnitude of the TGF-β1-dependent events associated with myofibroblast conversion is markedly dependent on TRPV1 activity. Inhibiting this membrane receptor protein with CPZ and/or intracellular events induced by its activity may provide novel approaches for the modulation of myofibroblast activity so as to prevent or ameliorate scarring.

**Figure 12:**
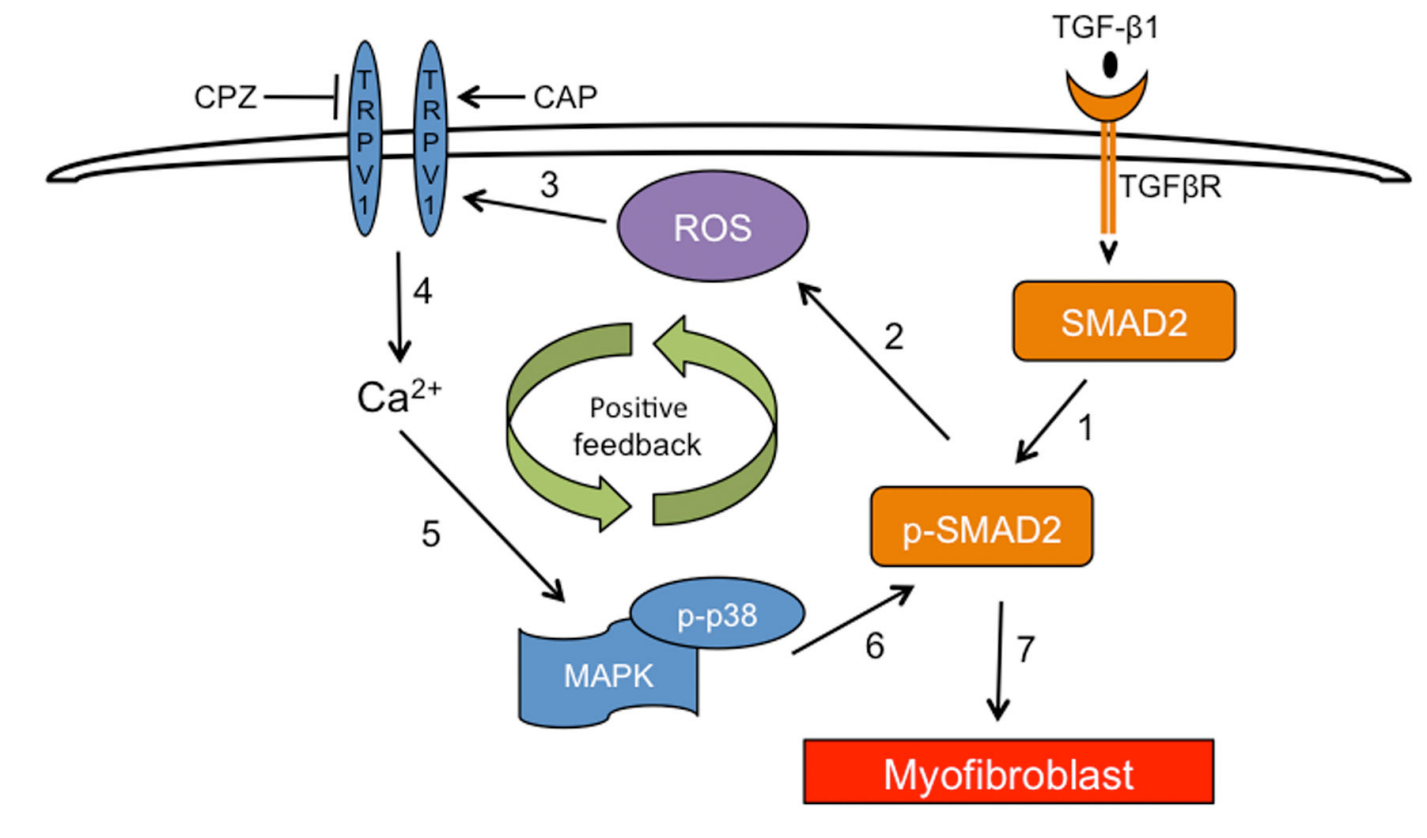
Summary model illustrating dependence of TGFβ on TRPV1 to induce myofibroblasts. TRPV1 potentiates TGF-β1-induced α-SMA expression through a positive feedback loop that enhances and prolongs SMAD2 activation. The following steps have been reported and/or have been revealed in this study: 1) TGF-β1 activates TGFβ receptors and in turn the latter phosphorylate SMAD2; 2) this activated SMAD enhances the cellular ROS generation rate through a NOX-dependent process  3 and 4); ROS activates TRPV1 leading to calcium influx; 5) the increased [Ca2+]i contributes to rapid phosphorylation of MAPKs which is short lived for ERK1/2 and JNK1/2 but extended for p38; 6) activated p38 contributes to further phosphorylation of SMAD2. Repeated cycling through this loop results in an enhanced and long- lived SMAD2 activation that; 7) induces myofibroblast formation.

The results point to the existence of a complex set of intracellular interactions that establish a positive feedback mechanism aimed at prolonging the activated state of SMAD2, the canonical mediator of TGFβ cell signaling (Figure 12). On the one hand, inhibition of TRPV1 reduced the p-SMAD2 level attained 30 min after TGF-β1introduction by about 55% (Figure 3). This reduction resulted in the end in an even larger, 70% reduction in the amount of α-SMA accumulated after 3 days (Figure 4). Not surprisingly, the highly reduced level of accumulated α-SMA was not sufficient to allow for the development of the full myofibroblast in culture (Figure 2) and reduced the amount of α-SMA staining in organ culture (Figure 1). On the other hand, tracking the extracellular calcium dependent and CPZ-, and TRPV1 siRNA-sensitive [Ca^2+^]_i_ inhibitory effects demonstrated that TGF-β1and/or p-SMAD2 are directly or indirectly fully responsible for the activation of TRPV1 (Figure 5). The result will be a seemingly endless cycle where TGF-β1-activated p-SMAD2 activates TRPV1 and in return TRPV1 activates SMAD2. The relatively slow rate of the evoked [Ca^2+^]I rise, vis. a vis. the rapidity of change when the response is generated by the TRPV1 agonist CAP (Figure 5), suggested to us that the TRPV1 transactivation may involve one or more intermediate steps dependent on an agent that undergoes a time dependent accumulation. The features of reactive oxygen species fit well this expectation. As observed in other cell types [32,33], activation of the TGFβ1R-SMAD2 system caused a gradual extended rise in the steady state level of cellular ROS (Figure 8 A and B). Abolishing this increase with two distinct ROS generation inhibitors, (i.e. DPI or NAC) caused very large reductions in the TRPV1-dependent Ca^2+^ influx (Figure 9) indicating the involvement of ROS in the receptor channel transactivation. We have indeed observed that addition of H_2_O_2_ (a chemical surrogate of ROS) to the HCF elicited Ca^2+^ transients, which were abolished by CPZ or extracellular calcium sequestration (not shown). In primary nociceptive neurons, exposure to oxidative stress markedly sensitizes TRPV1 via covalent modification of cysteines [34]. The mechanism in HCF through which ROS leads to TRPV1 activation remains to be elucidated.

The other branch of the current studies focused on the mechanism through which TRPV1 causes feedback activation of SMAD2. Following TGFβ addition, the levels of activated (phosphorylated) p38 underwent a continuous, completely TRPV1-dependent, large rise (Figure 6) for at least 16 h. This activation, in turn, mediated the TRPV1-dependent enhancement in p-SMAD2; both soon after addition of TGF-β1 or the following day, inhibition of p-p38 kinase activity caused p-SMAD2 level reductions similar to the declines observed (Figure 3) by inhibition of TRPV1 or its dependent calcium rise. Remarkably, though, ROS activity was also reduced when cells were preincubated with CPZ prior to introduction of TGF-β1 (Figure 8), indicating that the extent of ROS generation increase in the control condition was reinforced by mechanisms initiated by TRPV1 activation. All of these apparently reciprocal interactions have been summarized in the model for a positive feedback signal transduction loop (Figure 12) where we have incorporated preexisting knowledge about the cytosol to nuclear translocation of both p38 and SMAD2 following their activation. The feedback loop seems to be tuned to cause the observed progressive accumulation of ROS above the pre-activated level at the least, for the first 16 h after introduction of TGF-β1from 55% at 30 min to more than 300% at 16 h. This rising trend is in agreement with the observations made in lung, cardiac as well as kidney fibroblasts [7,35,36,37]. The persistent, TRPV1-driven activation of p38, leads in turn to a persistent or prolonged activation of SMAD2 that allows for the continued activation of the transcription factors that lead to accumulation of α-SMA and development of the myofibroblast phenotype over a 3 day period.

Previous studies on the effects of interactions between TRPV1 and TGFβ signaling on downstream events in other systems have yielded conflicting results. Supporting our findings, earlier studies on TRPV1 in odontoblasts and in sensory neurons showed that the TRPV1 signaling response is elicited through sequential activation of ERK1/2 MAPK and increases in expression of the transcription factor, Egr-1, leading to cyclin dependent kinase 5 (CDK5)-mediated TRPV1 phosphorylation, increases in Ca2+ influx and intracellular acidification leading to dental nociception [38,39]. On the other hand, TRPV1 activation by TGFβR suppresses progression of renal fibrosis caused by DOCA-induced salt dependent hypertension since in TRPV1 knockout mice fibrosis is reduced [40]. Similarly, in another study characterizing TRPV1 involvement in modulating the effect of TGFβR activation by myocardial infarction (MI) on survival, it was shown that TRPV1 has instead a protective role in the healing process by reducing fibrosis and improving contractile performance [41]. Thus there appears to be a tissue-specific involvement of TRPV1 in modulating TGFβ signaling and fibrotic healing, making our study important for vision research. Our study provides novel insight into how TRPV1 activation by TGFβR induces corneal scarring during wound healing. It elucidates a previously undiscovered link between TRPV1 activation and the generation of ROS. Furthermore, we show that myofibroblast development is dependent on activation of a positive feedback signaling system that results in sustained SMAD2 activation leading to downstream rises in α-SMA expression that can compromise corneal transparency. Taken together, suppressing injury-induced corneal TRPV1 activation by TGFβ may be a novel therapeutic option to selectively decrease fibrosis in a clinical setting.

In summary, we found that exposure of HCFs to TGF-β1 induces TRPV1 transactivation through SMAD2-mediated ROS formation resulting in a positive feedback loop that greatly augments the persistence of activated states for p38 and SMAD2. Our data indicate that, in the corneal fibroblast without this reinforcement mechanism, TGFβ cannot elicit a major conversion of fibroblasts into myofibroblasts and that in the whole wounded cornea the inhibition of the TRPV1 contribution is sufficient to essentially abolish the emergence of the myofibroblast phenotype during the healing response phase. This co-receptor transactivation biological strategy for augmentation of TGFβ effects may be operative in multiple other cell types, though it may involve other receptors or channels besides TRPV1. Disruption of such loops through suppressing member function provides a novel concept that requires further evaluation.
